# Impact of Diets on Cognitive and Clinical Outcomes in Alzheimer’s Disease: A Scoping Review

**DOI:** 10.7759/cureus.81367

**Published:** 2025-03-28

**Authors:** Quinn Jackson, Rachel Fricker, Erjola Toska, Alessandra Ottley, Alexa Carleo, Gabriella Cutrali, Olivia D'Alessio, Raquel Rossman, Samuel Kruchakov, Abraham Edelstein, Lubov Nathanson

**Affiliations:** 1 Neurological Surgery, Nova Southeastern University Dr. Kiran C. Patel College of Osteopathic Medicine, Fort Lauderdale, USA; 2 Neurology, Nova Southeastern University Dr. Kiran C. Patel College of Osteopathic Medicine, Fort Lauderdale, USA; 3 Institute of Neuro-Immune Medicine, Nova Southeastern University Dr. Kiran C. Patel College of Osteopathic Medicine, Fort Lauderdale, USA

**Keywords:** alzheimer’s, diet(s), fasting, outcomes, supplements

## Abstract

Alzheimer's disease (AD) is characterized by beta-amyloid plaques and neurofibrillary tangles, leading to damage of neuronal synapses, negatively impacting memory and cognition. Research efforts have shown the benefit of proper nutrition in reducing the progression of AD and slowing the condition's progression. Specifically, nutritional intake has been demonstrated to influence neuro-inflammatory pathways. Various diets, including ketogenic, Mediterranean, Mediterranean-DASH Intervention for Neurodegenerative Delay (MIND) diet, and Dietary Approaches to Stop Hypertension (DASH), have been shown to prevent the onset of AD to different degrees. The primary objective of this scoping review was to assess the impacts of various diets on AD onset and progression to identify unmet needs and gaps in understanding. Following PRISMA (Preferred Reporting Items for Systematic Reviews and Meta-Analyses) guidelines, peer-reviewed literature was searched using Boolean operators: “Alzheimer’s” AND “ketogenic” OR “DASH diet” OR “MIND diet” OR “Mediterranean diet” on EMBASE, MEDLINE, and Web of Science databases. The search was restricted to articles written in English and published in the United States between January 1, 2013, and September 30, 2023. The initial search yielded 121 articles after removing duplicates. After filtering based on inclusion and exclusion criteria, 24 articles were selected for further critical appraisal screening. The critical appraisal yielded 12 articles for the analysis included in this scoping review. This review supported the Mediterranean diet as the most effective in improving outcomes among patients with AD. The Mediterranean diet consists of a high intake of vegetables, fruits, legumes, nuts, olive oil, and unrefined cereals, a moderate intake of fish and wine, and a low intake of dairy products, meat, poultry, and saturated fat. Other diets reviewed, including the DASH and MIND diets, also have promising effects on AD, with less conclusive evidence when compared to the Mediterranean diet. Limitations, including small sample sizes, short durations, and socioeconomic constraints impacting compliance, were noted. This scoping review supports the need for dietary recommendation guidelines for patients with AD.

## Introduction and background

Alzheimer's disease (AD) is characterized by beta-amyloid plaques and neurofibrillary tangles [1,2]. Both plaques and neurofibrillary tangles are dense filaments that are highly insoluble, causing destruction and damage to neuronal synapses, leading to an impact on memory and cognition [3]. AD is a gradual and progressive neurodegenerative process, typically manifesting after the age of 65, referred to as late-onset AD, or, less commonly, before the age of 65, referred to as early-onset AD [4]. AD is a multifactorial and heterogeneous process; for example, Trisomy 21 is considered a risk factor for developing early-onset dementia [4]. The symptoms and different levels of cognitive impairment or disability are used to classify the stage of AD. The stages are the preclinical or presymptomatic stage, mild cognitive impairment, and dementia stage. The dementia stage is further divided into mild, moderate, and severe stages [4].

AD is the sixth leading cause of death in the United States, leading to research efforts, especially as it relates to the underlying pathophysiology as well as preventing the onset and delaying the progression of the disease. This includes a focus on identifying neuroprotective diets and vitamins that can be utilized for preventing or delaying AD onset and progression [5]. It is well-documented that nutrition can modulate the immune system and influence neuro-inflammatory pathways [6-9]. Research efforts have shown the benefit of proper nutrition in reducing the progression to AD. Specifically, nutritional intake has been demonstrated to influence neuro-inflammatory pathways. Among various studies on AD, researchers have aimed to identify the relationship between nutrition and AD. Such studies have evaluated the link between the ketogenic diet [6,10,11], Mediterranean diet [6,12], DASH (Dietary Approaches to Stop Hypertension) diet [6,12], plant-based diets [13], and AD onset and progression. The ketogenic diet is characterized by a low carbohydrate and high fat intake diet [14]. The Mediterranean diet consists of a high intake of vegetables, fruits, whole grains, legumes, and nuts, a moderate intake of fish, poultry, and red wine, and a low intake of red processed meats with olive oil [6]. The DASH diet consists of recommending a high intake of fruits, vegetables, nuts, and wholegrain products with greater emphasis on low-fat dairy foods and low dietary sodium and does not recommend alcohol [6]. The MIND (Mediterranean-DASH Intervention for Neurodegenerative Delay) diet is a hybrid of the Mediterranean-DASH diet that is designed with dietary components and servings linked to neuroprotection and dementia prevention [15]. The MIND diet emphasizes natural plant-based foods with a stronger emphasis on the consumption of berries and green leafy vegetables and limits intakes of animal and high saturated fat foods [15]. In addition to diets, the use of anti-inflammatory and neuro-protective supplements such as probiotics [9], medium-chain triglycerides (MCT) oil [10,11], omega-3 fatty acids, polyphenols, and antioxidants [6,8] have been evaluated for their benefits to patients in preventing the onset and progression of AD. Some studies have also focused on specific vitamins, such as vitamin D, suggesting a deficiency may lead to neurodegenerative effects emphasizing AD [16]. There have also been animal studies that have shown that eating patterns alone, such as intermittent fasting, can play a preventative role in pathologies associated with memory decline seen in AD [17].

Currently, to date and to the best of our knowledge, there are no scoping reviews that comprehensively characterize the impact of nutritional intake and dietary consumption on AD [7,12,13,18]. Given the absence of clear dietary guidelines, a cumulative analysis of clinical impacts becomes essential to provide a foundation for developing evidence-based recommendations and further research efforts, especially as comparisons across different diets/vitamins remain limited [19].

Previous studies are often in the context of AD comorbidities such as type II diabetes [10,13] or specific pathogenic pathways like cognitive aging [6,12] and the gut-brain axis [9], as well as lifestyle factors like psychosocial influences, which play a significant role in onset and progression [13]. While a comprehensive understanding of diets that may be protective is needed, many factors have hindered these efforts. In one systematic study, researchers identified that the mean age of study participants posed a barrier in identifying an association with AD [18]. In addition, many of the diets being evaluated are in stark contrast to the standard American diet, which is known to contribute to many chronic diseases, including AD [13,20]. As such, our efforts focused exclusively on patients over 65 years of age residing in the United States. In addition, the scoping review included studies published within 10 years and excluded review studies, abstracts, posters, or studies not in English.

## Review

Methods

Inclusion/Exclusion Criteria

To meet inclusion criteria, articles were required to be peer-reviewed, written in English, published in the United States, and based on studies conducted between January 1, 2013, and September 30, 2023. Eligible studies had only human male and female participants, over 65 years of age, with a diagnosis of AD and without existing neurological comorbidities, including but not limited to Parkinson’s disease, Huntington’s disease, multiple sclerosis, or head trauma to exclude factors that remove the focus on clinical outcomes of AD patients.

There were no restrictions placed on the participants’ sex, race, ethnicity, or culture to broaden the scope and focus more generally on clinical outcomes. Studies were not excluded based on the country in which they were conducted, as long as the article was written in the English language. In addition, specific diets included in the search include Ketogenic, DASH diet, MIND diet, and Mediterranean diet. All systematic and scoping review articles were excluded. Publications for which full text was unavailable were also excluded.

Information Sources

The base search strategy involved the analysis of key terms in MeSH and from relevant articles in the following databases: EMBASE, Web of Science, and Ovid MEDLINE. The same databases were used for the full search strategy. All searches were run in September 2023, and the base search was adapted for each database.

Screening and Study Selection

**Figure 1:**
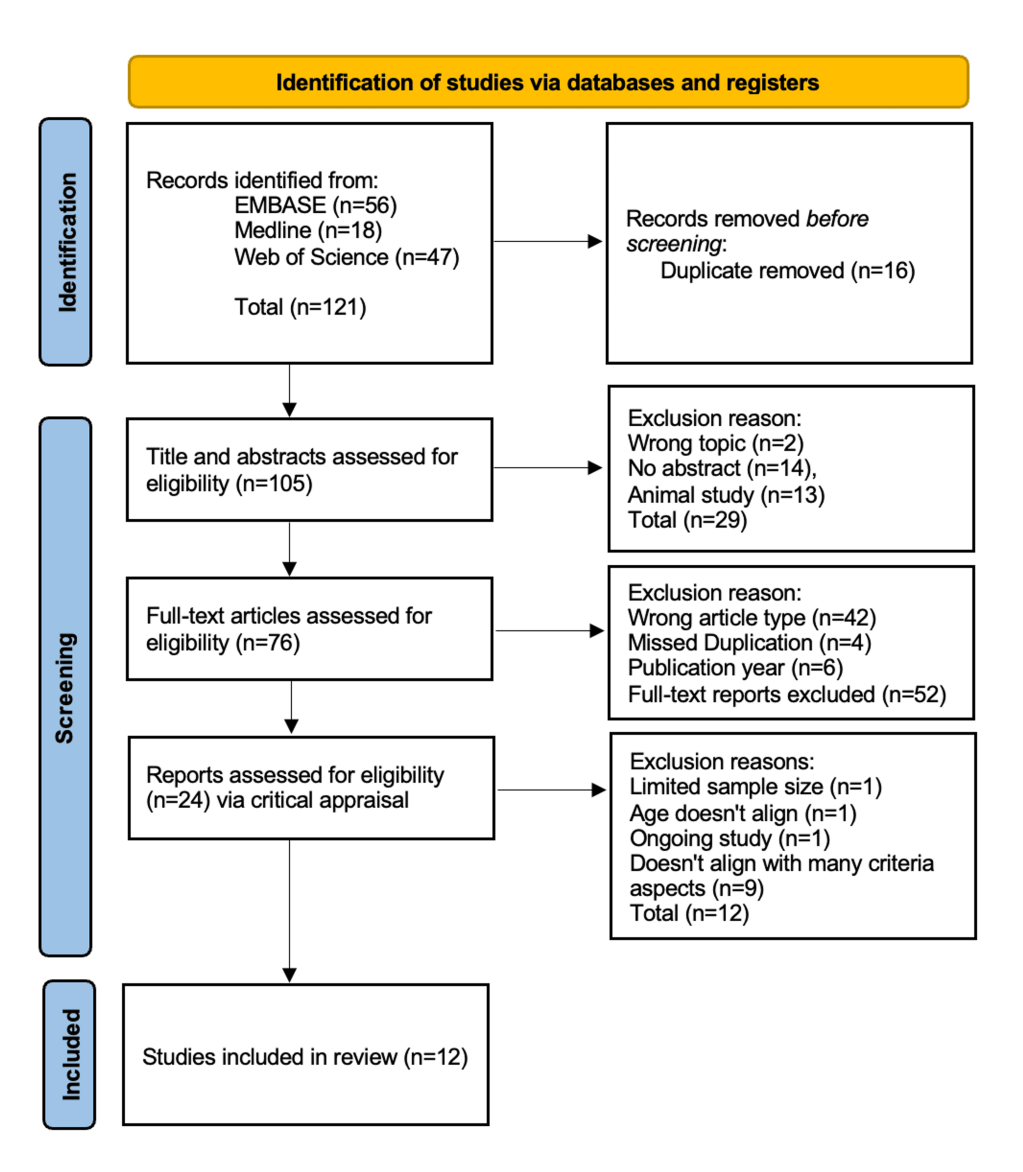
PRISMA flow diagram. PRISMA: Preferred Reporting Items for Systematic Reviews and Meta-Analyses.

The search identified 56 articles in EMBASE, 18 articles in MEDLINE, and 47 articles in Web of Science. This resulted in a total of 121 articles assessed. After duplicates were removed, 76 articles were kept for further review. Out of these 76 articles, 52 articles were removed because they were either considered a review article, an animal study, or not written in English. As a result, 24 studies were retained for analysis, and after analysis, 12 articles met all the inclusion criteria and were included. This screening and selection process is depicted using the Preferred Reporting Items for Systematic Reviews and Meta-Analyses (PRISMA) flowchart illustrated in Figure 1.

Search Strategy

The research question was based on the Population, Concept, and Context (PCC) strategy, establishing Population for men and women over the age of 65 years and older with existing AD and no other neurological comorbidities, Concept for the clinical outcomes of different neuroprotective diets as it relates to AD’s progression and Context for any article published during 2013 to 2023 in the United States. Based on these definitions, the guiding question was, “What literature exists regarding the effect of diet and/or nutritional factors on the onset and progression of AD in patients over 65 years old living in the United States, and are there any gaps in this knowledge?” Data collection occurred on September 12, 2023, and searches were conducted in EMBASE, MEDLINE, and Web of Science. The choice of databases resulted from the high number of relevant primary health articles available. Two reviewers conducted the research independently, using controlled descriptors from the Medical Subject Headings (MeSH): “Alzheimer’s,” “ketogenic,” “DASH diet,” “MIND diet,” and “Mediterranean diet.” The search terms and results from EMBASE, Ovid Medline, and Web of Science can be seen in Tables 1-3, respectively.

**Table 1:** Search criteria for EMBASE and results (n=56).

Search	Keywords/subjecting heading terms	Results hits
#1	'dash diet':ab,ti,kw or 'mind diet' or 'mediterranean diet':ab,ti,kw or 'ketogenic diet':ab,ti,kw	18,535
#2	Alzheimer's disease:ab,ti,kw	29,065
#3	1 and 2	74
#4	3 AND [age]/lim AND ‘human’/de with 2013-2023 years	56

**Table 2:** Search criteria for Ovid Medline and results (n=18).

Search	Keywords/subjecting heading terms	Results hits
#1	'dash diet':ab,ti,kw or 'mind diet' or 'mediterranean diet':ab,ti,kw or 'ketogenic diet':ab,ti,kw	438
#2	Alzheimer's disease:ab,ti,kw	340,009
#3	1 and 2	195
#4	3 AND [age]/lim AND ‘human’/de with 2013-2023 years	18

**Table 3:** Search criteria for Web of Science and results (n=47).

Search	Keywords/subjecting heading terms	Results hits
#1	'dash diet':ab,ti,kw or 'mind diet' or 'mediterranean diet':ab,ti,kw or 'ketogenic diet':ab,ti,kw	1,772
#2	Alzheimer's disease:ab,ti,kf	180,689
#3	1 and 2	124
#4	3 AND [age]/lim AND ‘human’/de with 2013-2023 years	47

For this research effort, publications were reviewed on the subject of AD patients consuming various nutrition plans, including the ketogenic, DASH diet, MIND diet, and Mediterranean diet, with an emphasis on clinical outcomes such as cognition and memory. The PRISMA method was adopted to systematize the inclusion process of the studies.

Selection of Sources of Evidence

To achieve a sufficient level of consistency in the screening process, each reviewer screened the same 76 articles and discussed the results. They also discussed any changes that needed to be made to the screening and data extraction plan. Four reviewers working in pairs evaluated the titles, abstracts, and full text of all publications for potentially relevant publications. Disagreements among reviewers regarding study selection were resolved through discussion until a consensus was reached, with other reviewers consulted on an as-needed basis. 

Data Charting Process

Data was extracted and charted from the articles by two or more independent reviewers using an Excel (Microsoft Corp., Redmond, WA) spreadsheet. The draft data extraction tool was modified and revised as necessary while extracting data from each included evidence source. The two reviewers independently charted the data for their assigned articles. The results were discussed among the two reviewers, and any disagreements that arose were resolved through discussion and, if applicable, other reviewers. The Excel sheet was continuously updated in an iterative process.

Critical Appraisal of Individual Sources of Evidence (Quality Assessment)

The articles gathered by the Tier II Data Extraction were appraised via the Joanna Briggs Institute (JBI) Critical Appraisal Tool [21]. The critical appraisal tool has the ability to reveal possible extrapolation of data, and it improves the quality of articles in this scoping review. Most importantly, the JBI Critical Appraisal Tool assists researchers in excluding articles that have varying degrees of biased discoveries/conclusions. The tools allowed to classify the articles as high risk of bias (scores less than 50%), moderate risk of bias (scores between 50% and 70%), and low risk of bias (scores above 70%).

After the selection of 76 publications, these publications were aligned with the inclusion criteria. Then, based on the type of research being performed, they were matched with the appropriate checklist from the JBI Critical Appraisal Tool to help verify their reliability and unbiased opinions. Two researchers then discussed their findings, the appraisal score that they gave for each article, and the reason for inclusion or exclusion from the study. Twelve final articles were selected to be included in this manuscript since they met all inclusion criteria and were found to be reliable, unbiased articles based on the JBI Critical Appraisal Tool.

Results

Characteristics of Studies

Out of 12 included studies, 10 took place in the United States [14,15,22-29], one in Spain [30], and one in Germany [31]. Six of the 12 studies had sample sizes greater than 100 individuals, ranging from 102 participants to 923 participants [15,22,27,29-31]. Three studies contained approximately 20 participants [14,26,28], two contained approximately 75 participants [23,24], and one was a case study presentation [25].

Several different types of studies were conducted in order to evaluate the nutritional effects of AD in an older population (>65 years old) [5,12-32]. Among the studies included were a medical record search [30], longitudinal studies [15,27,29,31], cross-sectional studies [23,24], randomized control/cross-over studies [14,22,26], a case study report [25], and a pilot study [28].

The duration of each study spanned from distinct measurements (e.g., one brain scan) to 22 years. Three studies included in this scoping review obtained information from participants for over one year [15,27,30]. One study required self-reporting data for one year (surveys answered based on one year of information) before measurements were taken [23]. Seven of the studies involved dietary interventions that followed patients over the course of several weeks, ranging from six weeks to 52 weeks [14,22,24-26,28,31]. One study was conducted for an unknown amount of time. Yearly data from each patient was obtained until brain autopsy results were eventually able to be obtained [29].

Key Findings 

The 12 studies evaluated and assessed the effects of various diets (the Mediterranean diet, the Ketogenic diet, the DASH diet, and the MIND diet) and other lifestyle interventions on the development and/or progression of AD or AD-like symptoms. Three studies concluded that adhering to a Mediterranean diet reduced the risk of developing AD [15,27,30]. Adhering to a Mediterranean diet was also associated with better brain functioning, which was observed by less amyloid plaques and brain atrophy, as well as increased learning and memory [23,29,31]. Notably, in a study, it was found that mediotemporal volume accounted for 40% of the relationship between Mediterranean-like diet adherence and memory performance, suggesting that the diet's protective effects on memory may be partly due to its impact on mediotemporal brain structures [31]. This also was seen with brain gray matter volume in the right parahippocampal gyrus and right hippocampus, which showed a significant positive association with a Mediterranean-like diet [31]. These findings reinforce the Mediterranean diet's role as a protective factor against memory decline and mediotemporal atrophy, likely through its ability to reduce amyloid and tau pathology [31]. The temporal region of the brain, crucial for memory processing, benefits from this reduction in neuroinflammation [30].

In one study, when the MIND diet and/or DASH diet were highly adhered to, there was an associated decreased risk of developing AD [15]. In another two studies, the Modified Mediterranean diet and the Mediterranean diet with exercise were shown to improve overall brain health and spatial working memory [14,22]. In addition, another study found that patients diagnosed with AD had lower adherence to adequate exercise and the Mediterranean diet as compared with their healthy counterparts [24]. Furthermore, researchers in another study found that patients with AD who followed a Ketogenic diet had an increased quality of life (based on the participants' own subjective meaning of that relating to daily happiness) as compared to individuals with AD who did not follow a Ketogenic diet [26]. The compliance of a Ketogenic diet also proved to benefit individuals at risk of developing AD by increasing cerebral perfusion and displaying less cognitive decline [28]. The Ketogenic diet was also shown to restore cognitive function in individuals with mild AD who are apolipoprotein4 (APOE4) positive [25]. The key characteristics of the included sources are summarized in Table 4.

**Table 4:** Table of included source of evidence characteristics. rMED: Relative Mediterranean Diet Score, MRI: magnetic resonance imaging, CSF: cerebrospinal fluid, SUCCAB: Subtests of the Cognitive Assessment Battery, PGI-AD: Polygenic Index for Late-Onset Alzheimer’s Disease, MEDAS: Mediterranean Diet Adherence Screener, MoCA: Montreal Cognitive Assessment, ACE-III: Addenbrooke's Cognitive Examination, 3rd edition, ADCS-ADL: Alzheimer's Disease Cooperative Study Activities of Daily Living, QOL-AD: quality of life in Alzheimer's disease, HbA1C: hemoglobin A1C, HDL: high-density lipoprotein, LDL: low-density lipoprotein, MIND: Mediterranean-DASH Intervention for Neurodegenerative Delay, DASH: Dietary Approaches to Stop Hypertension.

Authors	Aim of study	Country of origin	Study setting	Methods/type of study	Main findings	Limitations
Andreu-Reinón et al. (2021) [30]	To assess the protective role of the Mediterranean diet on the development of Dementia in healthy individuals	Spain	16,160 participants ranging from 30 to 70 years old, who enrolled in the study between 1992-1996. 459 participants were dementia cases, where 265 were female, and 194 males	Medical Record Search: The evaluation of participants was conducted using rMED (relative Mediterranean Diet score) compared to a two-phase validation protocol by physicians to assess cases of Dementia	High adherence to the Mediterranean diet was associated with a 20% lower risk of Dementia development and an 8% lower hazard ratio for developing Dementia when following a continuous Mediterranean diet. The association was statistically significant in women but not for men. Participants with lower education were found to have a stronger association	Medical record search, so there are limitations to the conclusion that they could draw; Length of study = 22 years
Ballarini et al. (2021) [31]	To discover if AD and cognitive function are related to following a Mediterranean diet	Germany	512 participants; participants had an average age of 69.49 ± 5.86 years, and 270 participants were female. Participants included a range of cognitively normal individuals and those at higher risk for developing AD	Longitudinal Cross Section Study: evaluation of participants included cognitive assessment, dietary assessment, MRI acquisition, CSF sampling, Voxel-Based Morphometry Analysis	A positive correlation between memory functioning and adherence to the Mediterranean diet was found. One main finding observed was the preservation of the temporal regions in the brain, which suggests the preservation of memory function. The Mediterranean diet is associated with less amyloid pathology and less brain atrophy	The study does not allow for causal inference in participants. Additionally, adherence to the diet is based on sample medians and dietary guidelines and not specifically the consumption of beneficial foods. Length = median 52 weeks
Brinkley et al. (2022) [14]	To evaluate older adults at risk for developing AD (based on association with CSF biomarkers) when analyzing the effects of the Modified Mediterranean Ketogenic diet compared to an American Heart Association diet	United States	20 participants, 64.3 ± 6.3 years, 75% female and 25% male	Random Cross-Over Trial: for 6 weeks, half of the participants ate a Mediterranean Ketogenic diet, and half of the participants ate an American Heart Association diet. After 6 weeks on each diet, there was a 6-week break, and then participants switched diets and ate them for another 6 weeks.	Individuals who follow a Modified Mediterranean Ketogenic diet showed improvements in body composition and body fat, which thus has a large impact on brain health	The study includes a small sample size, a short duration of the study, and a lack of correction for multiple comparisons. Length = 18 weeks
Hardman et al. (2020) [22]	To assess the effect of a Mediterranean diet, exercise, and both on health, cognition, and memory on older persons living independently in aged-care communities	United States	102 participants between the age of 60 and 90 years old started in the study with 29 males and 73 females. After 6 months only 81 participants, 24 males and 57 females, remained in the study	Randomized Control Trials: The primary outcomes of the study included memory response times using SUCCAB memory tests. Secondary outcome measurements included blood pressure, arterial stiffness, and inflammation biomarkers	Participants who followed the Mediterranean diet and exercised showed significant improvements in spatial working memory	There was limited funding, which led to restraints on supporting participants throughout the study; Length = 6 months
Karstens et al. (2019) [23]	To examine the effect of the Mediterranean diet on cognition and brain structural integrity on the development of AD and Vascular Dementia	United States	82 participants, age ~ 68.8 years old, 50% male and 50% female	Cross-Sectional Study: participants reported adherence to a Mediterranean diet, and effects were evaluated using Brain MRIs	Participants with higher adherence to the Mediterranean diet demonstrated better learning and memory and larger dentate gyrus volumes	Potential low power in detecting associations between the Mediterranean diet and neuroimaging due to the small sample size.; Length = self-reported dietary assessment over 1 year and then one scan
Mamalaki et al. (2024) [27]	To determine whether genetic risk factors may moderate the association between adherence to the Mediterranean diet and AD incidence	United States	537 participants without dementia or AD at baseline	Cohort/Longitudinal Studies: A Polygenic Index for late-onset AD (PGI-AD) was used to measure the effect of the Mediterranean diet on the development of AD. A 3-year follow-up interval for this study	Participants who had low adherence to the Mediterranean diet had a tenfold increase in developing AD over those participants who had a high adherence to the Mediterranean diet	The follow-up period of 3 years was short to determine the cause and causality. Length = 3 years
Martín et al. (2018) [24]	Evaluate the effect of AD on lifestyle habits, food, BMI, and loss of gustatory function	United States	75 patients with AD (mean age 77.5 years) and 267 healthy volunteers (mean age 73 years). Ages ranged from 65 to 96 and 86 participants were male and 237 female	Cross-sectional, observational, descriptive, and comparative cohort study: anthropometric measurements (lower BMI and weight) and MEDAS score.	Patients with AD demonstrated worse outcomes in anthropometric measurements, diet, exercise, and gustatory function when compared to the control group	A limitation is that dietary intake is affected by an individual’s health status and social background. Length = 6 weeks
Morrill & Gibas (2019) [25]	To determine if there is a nutritional intervention to prevent/treat AD	United States	71-year-old female	Case Study Report: measurements of triglycerides, insulin, HgAlC, ketones, and cognition using the MoCA (Montreal Cognitive Assessment)	Individuals who are APOE4 positive who follow a ketogenic diet may restore cognitive function in those with mild AD	This is a case study report so the sample size is limited to one person. Length = 10 weeks
Morris et al. (2015) [15]	To evaluate and compare the effects of the MIND diet, the DASH diet, and Mediterranean diet on the risk of developing AD	United States	923 participants (ages 58-98 years old) living in retirement communities and senior public housing in Chicago area	Longitudinal Study: participants were followed on average for 4.5 years. Diets were assessed using a semiquantitative food frequency questionnaire	High adherence to the MIND Diet, DASH Diet, and Mediterranean diet may reduce the risk of developing AD. The risk of developing AD may be reduced risk in individuals who moderately adhere to the MIND diet	This observational study assumes a causal effect between diet and AD. Limited information was provided on the food frequency questionnaire. Length = 4.5 years
Neth et al. (2020) [28]	To determine the effects of a Modified Mediterranean Ketogenic diet and American Heart Association diet on biomarkers, neuroimaging, and cognition in those at risk for AD	United States	20 participants (5 male and 15 female); subjective memory complaints (n = 11) or mild cognitive impairment (n = 9)	Pilot Study: measurements included Mini-Mental State Examination Score, CSF Biomarkers	Individuals at risk for AD who follow a ketogenic diet may benefit from less cognitive decline	This study has a small sample size. Results are susceptible to metabolic carry-over effects and difficulty with compliance to the diet. Length = 18 weeks
Phillips et al. (2021) [26]	To evaluate whether a 12-week modified ketogenic diet improved daily function, cognition, or quality of life in a hospital clinic of AD patients	United States	26 participants (16 male and 10 female) ranging between 50 to 90 years old, had dementia with a severity rating scale score < 19, a body mass index > 18.5, and a cohabiting trial partner willing to partake in a ketogenic diet	Randomized Crossover Trial: within-individual changes in cognition (ACE-III), daily function (ADCS-ADL), and quality of life (QOL-AD). Measurements also included weight, body mass index, HbA1C, triglycerides, HDL, LDL, and total cholesterol	Patients with AD may have improvement in daily function and quality of life by following a Ketogenic diet	The sample size and duration were small. Additionally, some patients may not have had AD due to trying to differentiate AD from other forms of Dementia. Length = 12 weeks
Yasar (2023) [29]	To determine the relationship between the Mediterranean diet and the Mediterranean-DASH Intervention for Neurodegenerative Delay (MIND) diet, specific food components, and beta-amyloid and tau protein accumulation, using brain from deceased people	United States	581 participants who had their diet information obtained and eventually brain autopsy results	Longitudinal Study: participants were followed and had their diet information collected until they passed away and then had a brain autopsy performed	It was observed that there was less buildup of abnormal proteins (especially beta-amyloid protein) seen in patients who adhered to a Mediterranean and/or MIND diets, as well as those who increased the intake of green and leafy vegetables	This study included a primarily white population, and it needs to be conducted on a more diverse population. Length = yearly information obtained from each participant (unknown amount of time) until eventual brain autopsy results

Gaps in Research

Five of the studies contained small sample sizes and consequently had a low level of confidence when stating their findings [14,23,25,26,28]. Two of the studies stated that the duration of the study and follow-up time was shorter than desired for determining causality [14,27]. Some of the studies had limited funding and limited compliance with dietary guidelines due to socioeconomic limitations [22,24]. Various confounding factors, such as metabolic carry-over effects [31] and a lack of diversity in the sample populations [29], could affect the validity of the findings in each study.

Discussion

The primary objective of this scoping review was to assess the impacts of various diets on AD onset and progression to identify unmet needs and gaps in understanding. This assessment was executed using an evaluation of the quality and quantity of literature available in popular research databases in English. This study also aimed to provide insight for future research on AD by identifying gaps in available knowledge and research. After following a strict protocol and performing thorough data extraction and analysis, the researchers can conclude that both objectives were successfully met.

Of the 121 initial search results, 24 articles met all other criteria standards. Meanwhile, 12 of the 24 articles were excluded after critical appraisal due to the lack of internal quality per our quality evidence assessment protocol. This shows a clear quantitative and qualitative deficiency of research addressing clinical outcomes of AD in older patients (>65) when therapeutic diet interventions were utilized, despite the aging population and increasing prevalence of AD in the United States. In addition, multiple studies admitted confidence difficulty due to small sample size, short lengths of study, and lack of diversity. Beyond these limitations, several themes emerged, revealing gaps in knowledge and unmet needs in the literature.

The Neglected Diets

Of the 12 studies included, the Mediterranean diet was studied most often, being a variable in nine of the 12 studies included. The ketogenic diet was the primary focus of three of the 12 studies included, while the DASH diet and the MIND diets were only considered in one paper each in this study. No studies that measured fasting or supplement usage in AD clinical outcomes were eligible for the final study extraction despite being included in the initial search. This furthered the growing research gap on this topic. The narrow scope of the studies makes the development of treatment guidelines for AD prevention and/or treatment challenging.

Quality of Life

There was a strong correlation between all neuroprotective diets featured in each study and positive clinical outcomes. Positive outcomes were focused on improved cognitive function and memory and were reported in seven out of the 12 papers included. The decline in quality of life is a major concern for patients with AD and their families, so research aimed at improving this variable has the potential for high social impact, thus warranting more research.

One publication found that patients with AD had more difficulty with diet adherence compared to their healthy counterparts due to several factors [27]. Despite the ketogenic diet having some support in literature as beneficial, it is typically not recommended by physicians due to poor patient compliance with preparing their meals at home without the supervision of a dietitian [28].

Two publications extracted in this scoping review were hindered by socioeconomic factors [22,24]. Further research focused on improving quality of life and socioeconomic factors can help policymakers and practitioners develop a holistic guide to AD treatment, which may increase diet adherence.

Disease Pathology

Cerebral perfusion and beta-amyloid plaques are theorized to play a key role in the pathogenesis of AD. In addition, APOE4 is a known allelic risk factor for AD development, and there is suspicion that it plays a role in beta-amyloid regulation [25]. Looking into gender-related differences of amyloid plaques, a study noted that women who consumed one glass of wine per week had lower amyloid plaques, whereas those who ate more red and processed meat had more amyloid plaques [29]. Men in this study who drank between one glass of wine per month and six glasses per week and had higher fish intake had lower amyloid plaques [29].

The Mediterranean diet, in association with progression to AD, showed a slower transition from mild cognitive impairment to AD [24]. However, this scoping review revealed that there is a lack of literature connecting diet to direct clinical outcomes measured concerning our current understanding of disease pathology. Further insight into this area would allow practitioners to finely tune diet recommendations to modulate AD pathogenesis.

The Research Question

This scoping review was centered around answering the following research question: What literature exists regarding how diet and/or nutritional factors affect the onset and progression of AD in patients over 65 years old living in the United States, and what gaps exist in this research?

Our scoping review revealed that 10 of the 12 studies included showed improved brain function and lifestyle changes [14,15,22,23,26-28,30,31]. Six of the 12 studies were based on patients with AD, and one was based on patients at risk of developing AD [15]. In addition, the findings from various sources contribute to our understanding of diet and AD from a holistic perspective. A large number of publications discussed the findings of one particular diet’s outcomes, neglecting the other relevant diets being researched. Our findings lead to a more cohesive understanding of the concrete evidence that relates directly to clinical outcomes of various diets for AD; however, many limitations regarding lifestyle outcomes warrant further investigations.

Implications for Future Research 

This scoping review demonstrates the need for further research on various diets and how they protect the brain and reduce AD pathogenesis. There is also a need for a comparison of various diets in the same experimental settings. Although this review has determined that the current literature shows a significant correlation between the Mediterranean diet and improved outcomes in patients with AD, there were many other diets whose effects have not been properly explored in the primary research. Future research must focus on the many alternatives to the Mediterranean diet to establish more comprehensive guidelines for managing AD. While this review focused solely on the positive effects of various diets on AD, future research should also consider how certain dietary factors may lead to worse outcomes. This expanded scope would likely help create a unique set of recommendations for AD patients regarding their diets.

Limitations

The limitations of this study relate to the review's methodology and the available research.

The methodology of this scoping review was limited by language and database bias. By conducting the initial search via EMBASE, Web of Science, and Ovid MEDLINE, articles unavailable on these engines would be missing from this study. In addition, limiting the inclusion criteria to studies published in English introduces potential bias. A level of subjectivity is to be expected in the data extraction process. Risk of bias evaluations were conducted using the JBI Critical Appraisal method. This scoping review assessed the currently available literature on neuroprotective diets and AD clinical outcomes. As a result, it would be inappropriate for use as sole evidence for clinical guidelines or recommendations in treating and/or preventing AD.

This scoping review was limited by the availability of literature relating to the clinical outcomes of AD in individuals over 65 years of age. Of the initial search results, only 12 studies met the inclusion criteria, addressed the initial research question, and lacked significant internal bias. The 12 studies included in this scoping review were found to be heterogeneous in study methodology, ranging from case studies to large-scale database searches, making standardized assessment challenging. These limitations may have influenced the comprehensiveness of the synthesized evidence.

Despite the previously discussed various limitations, this review still answers the review question at hand. Dietary factors likely play a role in the development and progression of AD in individuals older than 65 years of age. Most studies focused on the effects of the Mediterranean diet, while the other diets explored were the Ketogenic diet, DASH diet, and MIND diet. Overall, the clinical outcomes in the various studies displayed the positive effects of specific diets in treating AD. Meanwhile, more diverse and more studies with an increased number of patients and clear conclusive research is needed.

## Conclusions

In conclusion, this information is particularly useful for physicians in developing holistic treatment plans for their AD patients. Taking a dietary and lifestyle approach to managing the disease process is not only cost-effective, but also greatly improves a patient's quality of life. This scoping review also identified an unmet need in the field, as the need for more specific guidelines for dietary recommendations for AD patients is much needed and warranted.

Other diets reviewed, including the DASH and MIND diets also have promising effects on AD, albeit with less conclusive evidence. It is important to consider all the information gathered in this review to establish a unique set of dietary guidelines to provide patients with the best recommendations possible.
